# Find the Essence through the Phenomena: Cardiovascular Diseases and Biomarkers 2019

**DOI:** 10.1155/2020/1959364

**Published:** 2020-09-27

**Authors:** Shipeng Wei, Agata M. Bielecka-Dabrowa, Zhongjie Shi

**Affiliations:** ^1^MercyOne Des Moines Medical Center, Des Moines, USA; ^2^Medical University of Lodz, Lodz, Poland; ^3^Wayne State University, Detroit, MI, USA

This special issue is a continuous effort of our successful 2018 special issue to discover novel biomarkers in the risk prediction, screening, diagnosis, progression, and prognosis cardiovascular diseases. We are happy to see real-time advances in the field with different study methods in different stages of diseases.

## 1. Biomarkers in Myocardial Ischemia or Infarction

C. Fu et al. reported that low expression of bradykinin B2 receptor on circulating progenitor cells indicated the poor outcomes of myocardial infarction, based on data from 174 myocardial infarction patients. V. A. M. Goulart et al. were able to identify alterations in the glycerophospholipids, alpha-linolenic acid, and sphingolipid metabolisms in ST-segment elevation myocardial infarction patients, using ultra-high-performance liquid chromatography-tandem mass spectrometry and MS-based flow injection analysis. J. Zhao et al. reported by meta-analysis that circulating microRNA-499 can be used as a diagnostic biomarker for acute myocardial infarction. Q. Ouyang et al. studied the effects of apelin on left ventricular-arterial coupling and mechanical efficiency in rats with ischemic heart failure and found that rats with ischemic HF were characterized by deteriorated left ventricular mechanoenergetics. Apelin improves mechanical efficiency by the inhibiting cardiac fibrosis and apoptosis in left ventricular myocardium, reducing collagen deposition in the aorta and dilating the resistant artery. K. Pieszko et al. employed machine learning techniques and hematological markers in a robust enrollment of 5053 patients and found that neutrophil count and red cell distribution width have a strong association with all-cause mortality after acute coronary syndrome. R. Rajtar-Salwa et al. showed the time-synchronized relationship between ischemia and left ventricular dysfunction assessed by high-sensitive troponin I and N-terminal pro B-type NT-pro natriuretic peptide. M. Mocan et al. reviewed biomarkers of inflammation in left ventricular diastolic dysfunction, which had been previously associated with a heart failure mechanism.

## 2. Biomarkers in Heart Failure

P. Perge et al. found that vitamin D deficiency predicts poor clinical outcomes in heart failure patients undergoing cardiac resynchronization therapy. C.-H. Wang et al. showed the feasibility of amino acid-based metabolic profile in functional and prognostic assessment for heart failure outpatients. D. Simeunovic et al. showed the role of glutathione transferase P1 polymorphism in individual susceptibility to oxidative stress, inflammation, and endothelial dysfunction in coronary artery disease and idiopathic dilated cardiomyopathy. T.-L. Chuang et al. investigated the role of bone mineral density in the prediction of cardiovascular disease through atherogenic indexes in nonobese adults.

## 3. Biomarkers at a Specific Stage of Diseases

T. Fang et al. found that the preoperative serum CEA, CA125, and CA19-9 levels can help predict the resectability of cholangiocarcinoma. X. Liu et al. showed the preoperative abnormal changes in respiratory rate, TPTEF/TE, VPEF/VE, and lung compliance are indicative of the risk of postoperative pulmonary complications in infants with congenital heart diseases. W. Su et al. reported the use of integrated microfluidic device for enrichment and identification of circulating tumor cells from the blood of patients with colorectal cancer. M. Walentowicz-Sadlecka et al. found that placental soluble fms-like tyrosine kinase-1 (sFlt-1) and sFlt-1/25(OH)D ratio can be used as a diagnostic tool in gestational hypertension, preeclampsia, and gestational diabetes mellitus. I. Mozos et al. presented the links between high-sensitivity C-reactive protein (hsCRP) and pulse wave analysis in middle-aged patients with hypertension and high normal blood pressure (HNBP). Furthermore, vitamin D level, hsCRP, and low-density lipoprotein cholesterol provide valuable information in middle-aged hypertensive and HNBP patients, related to arterial stiffness and early arterial aging.

With so many novel and valuable biomarkers found and are being continuously found, we are closer to the essence of the phenomena and are more confident in the battle against cardiovascular diseases in the future.

*Shipeng Wei* *Agata M. Bielecka-Dabrowa* *Zhongjie Shi*

